# [^123^I]MIBG is a better early marker of anthracycline cardiotoxicity than [^18^F]FDG: a preclinical SPECT/CT and simultaneous PET/MR study

**DOI:** 10.1186/s13550-021-00835-1

**Published:** 2021-09-20

**Authors:** Alexandra Oudot, Alan Courteau, Mélanie Guillemin, Jean-Marc Vrigneaud, Paul Michael Walker, François Brunotte, Alexandre Cochet, Bertrand Collin

**Affiliations:** 1 Centre Georges-François Leclerc – Unicancer, Nuclear Medicine Department, Plateforme d’Imagerie et de Radiothérapie Précliniques, 1, rue Professeur Marion, BP 77980, 21 079 Dijon Cedex, France; 2 ImVIA, EA 7535, Université de Bourgogne-Franche-Comté, Dijon, France; 3 ICMUB, UMR CNRS 6302, Université de Bourgogne-Franche-Comté, Dijon, France

**Keywords:** Doxorubicin, Cardiotoxicity, Heart, [^18^F]FDG, [^123^I]MIBG

## Abstract

**Background:**

During anthracycline treatment of cancer, there is a lack for biomarkers of cardiotoxicity besides the cardiac dysfunction. The objective of the present study was to compare [^18^F]FDG and [^123^I]MIBG (metaiodobenzylguanidine) in a longitudinal study in a doxorubicin-induced cardiotoxicity rat model.

**Methods:**

Male Wistar Han rats were intravenously administered 3 times at 10 days’ interval with saline or doxorubicin (5 mg/kg). [^123^I]MIBG SPECT/CT (single photon emission computed tomography-computed tomography) and simultaneous [^18^F]FDG PET (positron emission tomography)/7 Tesla cardiac MR (magnetic resonance) imaging acquisitions were performed at 24 h interval before first doxorubicin / saline injection and every 2 weeks during 6 weeks. At 6 weeks, the heart tissue was collected for histomorphometry measurements.

**Results:**

At week 4, left ventricle (LV) end-diastolic volume was significantly reduced in the doxorubicin group. At week 6, the decreased LV end-diastolic volume was maintained, and LV end-systolic volume was increased resulting in a significant reduction of LV ejection fraction (47 ± 6% vs. 70 ± 3%). At weeks 4 and 6, but not at week 2, myocardial [^18^F]FDG uptake was decreased compared with the control group (respectively, 4.2 ± 0.5%ID/g and 9.2 ± 0.8%ID/g at week 6). Moreover, [^18^F]FDG cardiac uptake correlated with cardiac function impairment. In contrast, from week 2, a significant decrease of myocardial [^123^I]MIBG heart to mediastinum ratio was detected in the doxorubicin group and was maintained at weeks 4 and 6 with a 45.6% decrease at week 6.

**Conclusion:**

This longitudinal study precises that after doxorubicin treatment, cardiac [^123^I]MIBG uptake is significantly reduced as early as 2 weeks followed by the decrease of the LV end-diastolic volume and [^18^F]FDG uptake at 4 weeks and finally by the increase of LV end-systolic volume and decrease of LV ejection fraction at 6 weeks. Cardiac innervation imaging should thus be considered as an early key feature of anthracycline cardiac toxicity.

**Supplementary Information:**

The online version contains supplementary material available at 10.1186/s13550-021-00835-1.

## Background

The population of cancer survivors is rapidly growing resulting in a new medical need: the management of long-term complications of anticancer treatments in particular at the cardiovascular level [1–3]. Anthracyclines remain important drugs often mandatory in the treatment of many cancers despite their known cardiotoxicity. Anthracyclines mechanisms of action are multiple and still need to be better understood. They include DNA damage, apoptosis, inflammation, calcium dysregulation and reactive oxygen species production [4]. The recent European guidelines [5] recommend both baseline assessment and close monitoring of cardiac function to better prevent cardiotoxicity. Cardiotoxicity induced by anticancer treatment is today defined as any reduction of ejection fraction to below 50% or a > 10% reduction from baseline falling below the lower limit of normal [5]. There is thus no validated tool to detect cardiotoxicity before any change of cardiac function in clinical routine and that could be of interest in investigating cardioprotective strategies.

Different approaches were previously examined to detect cardiotoxicity before any drop in ejection fraction [6]. Nuclear imaging and magnetic resonance (MR) imaging are considered as promising tools to detect early cardiac toxicity. Moreover, nuclear imaging tracers allow to visualize molecular mechanisms involved in cardiotoxicity [2, 7]. [^123^I]metaiodobenzylguanidine (MIBG) cardiac uptake has been studied to evaluate cardiac adrenergic innervation [2, 7]. However, more studies are needed to clarify the role of this tracer. Indeed, initial steps in the progression of heart failure involve hyperadrenergic state that results in reduced neuronal reuptake and downregulation of adrenergic receptor [8]. [^123^I]MIBG imaging could therefore constitute a potential early marker for cardiac damage leading to heart failure.

More recently, [^18^F]fluorodeoxyglucose (FDG) has also been proposed as a positron emission tomography (PET) predictive tracer for cardiac toxicity [9–14]. PET imaging offers the advantage to allow more precise image quantification than single photon emission computed tomography/X-ray computed tomography (SPECT/CT) imaging in the clinical setting. Moreover, [^18^F]FDG PET imaging is a routine exam performed in most cancer patients. Its possible use for early detection of cardiac toxicity of anticancer treatments is therefore attractive because it would not require any additional exam and radiation exposure. However, literature data are scarce and somewhat contradictory, and the potential of [^18^F]FDG PET imaging to detect cardiac toxicity needs to be evaluated more precisely. It can be however hypothesized that modifications of cardiac [^18^F]FDG uptake should reflect several mechanisms that could be modified by anticancer treatments: cardiac tissue perfusion, inflammation or energy metabolism changes.

The objectives of the present study were to evaluate whether [^18^F]FDG and [^123^I]MIBG were able to detect early anthracycline-induced cardiotoxicity, before any modification of cardiac function. We used an experimental model of doxorubicin-induced cardiotoxicity in rats. Cardiac function was evaluated using MRI which can provide gold standard measurements of the left ventricular end-diastolic and end-systolic volumes. Imaging experiments using PET-MR and SPECT-CT were performed longitudinally in order to monitor in parallel and in the same animals the uptake of [^18^F]FDG, [^123^I]MIBG and cardiac function. This experimental design allowed to determine if the possible modifications of [^18^F]FDG or [^123^I]MIBG uptake occurred in the same time and are predictive of cardiac function impairment due to doxorubicin.

## Materials and methods

### Experimental model of doxorubicin-induced cardiotoxicity

All animal studies were conducted in accordance with the legislation on the use of laboratory animals (directive 2010/63/EU) and were approved by an accredited ethical committee (C2ea Grand Campus n°105) and the French ministry of research (authorization #6191). After baseline imaging, Wistar Han male rats (175–200 g, Charles River, France) were randomized into control (*n* = 5) or doxorubicin group (*n* = 5). Saline (control group) or doxorubicin (5 mg/kg doxorubicin Accord, Accord Healthcare—France) at 2.5 mL/kg intravenous injections were administered beginning on day 1 and repeated 3 times every 10 days (cumulative dose of 15 mg/kg). Imaging experiments were performed before the first doxorubicin/saline injection and every 2 weeks until the end of the 6-week experimental period. SPECT-CT and PET/MR imaging acquisitions were performed at 24 h intervals. The rats were observed daily, and body weights were monitored during the experimental period. No animals were excluded from the present study. One MR imaging dataset from doxorubicin group at week 2 was excluded because ECG-gating was not reliable.

### PET/MR imaging

Simultaneous PET/MR imaging was performed on a fully integrated system (MR Solutions, Guildford, UK) consisting of a 7 T dry magnet (Powerscan MRS-7024-PW) coupled to a SiPM-based dual ring PET system [15]. Animals from each experimental group were distributed all along the imaging experiment duration to reduce the potential bias.

Before anesthesia for PET/MR imaging, a blood sample was collected to measure glycemia (Novapro glucosemeter, Nova Biomedical, France). PET acquisitions were performed from 15 to 45 min after 20 MBq [^18^F]FDG intravenous injection (Curium, Dijon, France). The animals were not fasted before [^18^F]FDG injection, kept under anesthesia during the 15 min uptake time, and heated at 37 °C from anesthesia induction to the end of image acquisition. MRI cardiac cine acquisitions were performed simultaneously with PET acquisitions. Images were respiratory- and cardiac-gated (PC Sam, SAII, Stony Brook, USA). Twelve temporal frames per cardiac cycle were acquired. A spoiled gradient recalled echo-fast low angle shot (FLASH GRE) cine sequence was used with: repetition time: 10 ms, echo time: 3 ms, flip angle: 40°, slice thickness: 1.5 mm, voxel size: 0.23 × 0.23 × 1.5mm^3^ and 4 signal averages. The ventricle was covered by contiguous short axis slices from basis to apex. MR left ventricular volumes and mass were determined by manual contouring of left ventricle (LV) endocardium and epicardium on short axis slices (AnimHeart, CASIS, Dijon, France) on end-diastolic and end-systolic frames to determine end-diastolic volume (EDV), end-systolic volume (ESV), ejection fraction (EF) and LV mass. MR right ventricular volumes were determined by manual contouring of endocardium on short axis slices (ImageJ, NIH, USA) to determine right ventricular ejection fraction (RVEF). The investigator who performed cardiac function analyses was unaware of the treatment. PET reconstructions were performed for the complete 30-min scan. A 3D region of interest within LV tissue was manually delineated using VivoQuant software (Invicro, USA) and the radioactivity measured and corrected to LV mass (measured on MR images) and injected dose corrected to radioactive decay and expressed as a percentage of injected dose per gram (%ID/g).

### SPECT-CT imaging

SPECT-CT imaging was performed the day before PET/MR acquisitions. A Nanospect/CT plus camera (Mediso Ltd., Budapest, Hungary) was used. Rats were injected intravenously with 60 MBq [^123^I]MIBG (Adreview, GE Healthcare, Velizy-Villacoublay, France) 4 h before the beginning of the SPECT acquisition. X-ray CT acquisitions (55kVp, 34mAs) were performed first, followed by helical SPECT acquisitions with 70−110 s per projection frame. Iodine-123 photopeak (159 keV) was used with a 20% wide energy window. The CT and SPECT reconstructions were performed using an image processing software provided by Mediso Ltd. The SPECT/CT fusion image was obtained using Vivoquant software (Invicro, USA). Each scan was visually interpreted, and 3D regions of interest corresponding to the heart and mediastinum were manually drawn using VivoQuant software (Invicro, USA) in order to determine their radioactivity content (Additional file 1: Fig. S1). The cardiac region of interest was delineated manually using both SPECT signal and CT cardiac contours. The whole ventricular muscle and cavity were included in the region of interest. Mediastinum was defined as a fixed-size spherical region of interest at the level of the first intercostal space in front of trachea. Heart to mediastinum (H to M) ratio was calculated by dividing the Bq/mm^3^ in the myocardial region of interest by the Bq/mm^3^ in the mediastinal region of interest.

### Cardiac tissue processing: histomorphometry

At 6 weeks, animals were euthanized by an intraperitoneal injection of 140 mg/kg pentobarbital (Euthasol Vet®, Dechra Veterinary products). The heart tissue was collected, weighed after atrial tissue removal and paraffin-embedded for histomorphometry measurements after Hemalun-eosin staining. After slide scanning (Nanozoomer HT 2.0, Hamamatsu photonics K.K., Japan), the LV epicardial and endocardial contours were manually traced on short axis slices obtained in the central third of the heart. LV area (mm^2^) was determined, and the mean LV thickness (mm) was calculated as a ratio of the LV area over LV external contour length.

### Statistics

All statistical analyses were performed using the GraphPad Prism software. Data are expressed as mean ± SEM. The data were compared using a Student’s t-test (histomorphometry and heart weight) or a 2-way ANOVA followed by Bonferroni’s post-hoc tests (body weight, glycemia and imaging results). Correlations analyses were performed using Pearson correlation. P values under 0.05 were considered statistically significant.

## Results

### Physiological parameters

**Fig. 1:**
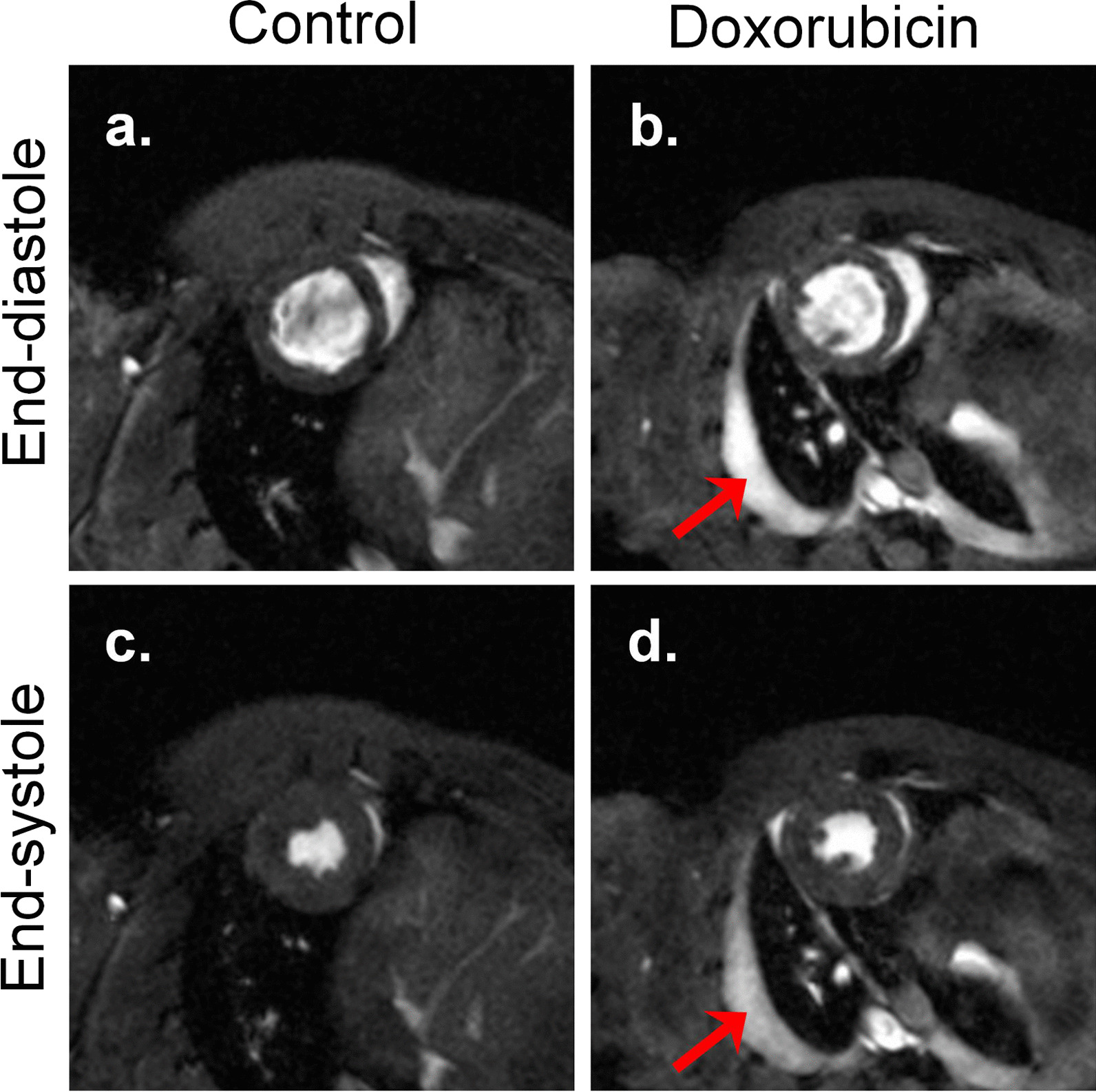
Representative MR images in short axis view. End-diastolic frame is represented on the upper panel and end-systolic frame on the lower panel. Left panel: MR images obtained in control rats. Right panel: MR images obtained in doxorubicin-treated rats. Red arrows indicate ascite/pleural effusion

During the 6-week experimental period, the impact of doxorubicin intravenous injections on general health status was not detectable up to week 6 except for body weight. Indeed, rat body weight evolution was significantly impacted by doxorubicin injections, starting from week 2 and persisting up to the end of the protocol (Table 1). Indeed, while body weight regularly increased in the control group, it remained at about the same level in doxorubicin-treated rats. During the last week of the protocol, two animals from the doxorubicin group developed ascite/pleural effusion. This may have participated to body weight increase between week 4 and week 6 in the doxorubicin group (Table 1) and could be observed on cardiac MR images at week 6 (Fig. 1b, d).

**Table 1:** Body/heart weights and glycemia measurements in vehicle and doxorubicin-treated rats

	Control	Doxorubicin	*p value*
*Body weight (g)*			
Baseline	222 ± 5	222 ± 4	*ns*
Week 2	274 ± 6	232 ± 3	*p* < *0.01*
Week 4	305 ± 9	208 ± 3	*p* < *0.001*
Week 6	322 ± 10	225 ± 14	*p* < *0.001*
*Left ventricle mass (mg)*			
Baseline	427 ± 22	423 ± 17	*ns*
Week 2	509 ± 20	413 ± 22	*p* < *0.01*
Week 4	492 ± 20	384 ± 9	*p* < *0.01*
Week 6	545 ± 21	446 ± 19	*p* < *0.01*
*Glycemia (mM)*			
Baseline	7.6 ± 0.3	6.9 ± 0.3	*ns*
Week 2	6.6 ± 0.2	6.9 ± 0.3	*ns*
Week 4	6.7 ± 0.2	6.7 ± 0.1	*ns*
Week 6	6.6 ± 0.2	6.0 ± 0.6	*ns*

*ns* not significant. Results are expressed as mean ± SEM

### Cardiac function by MRI

**Fig. 2:**
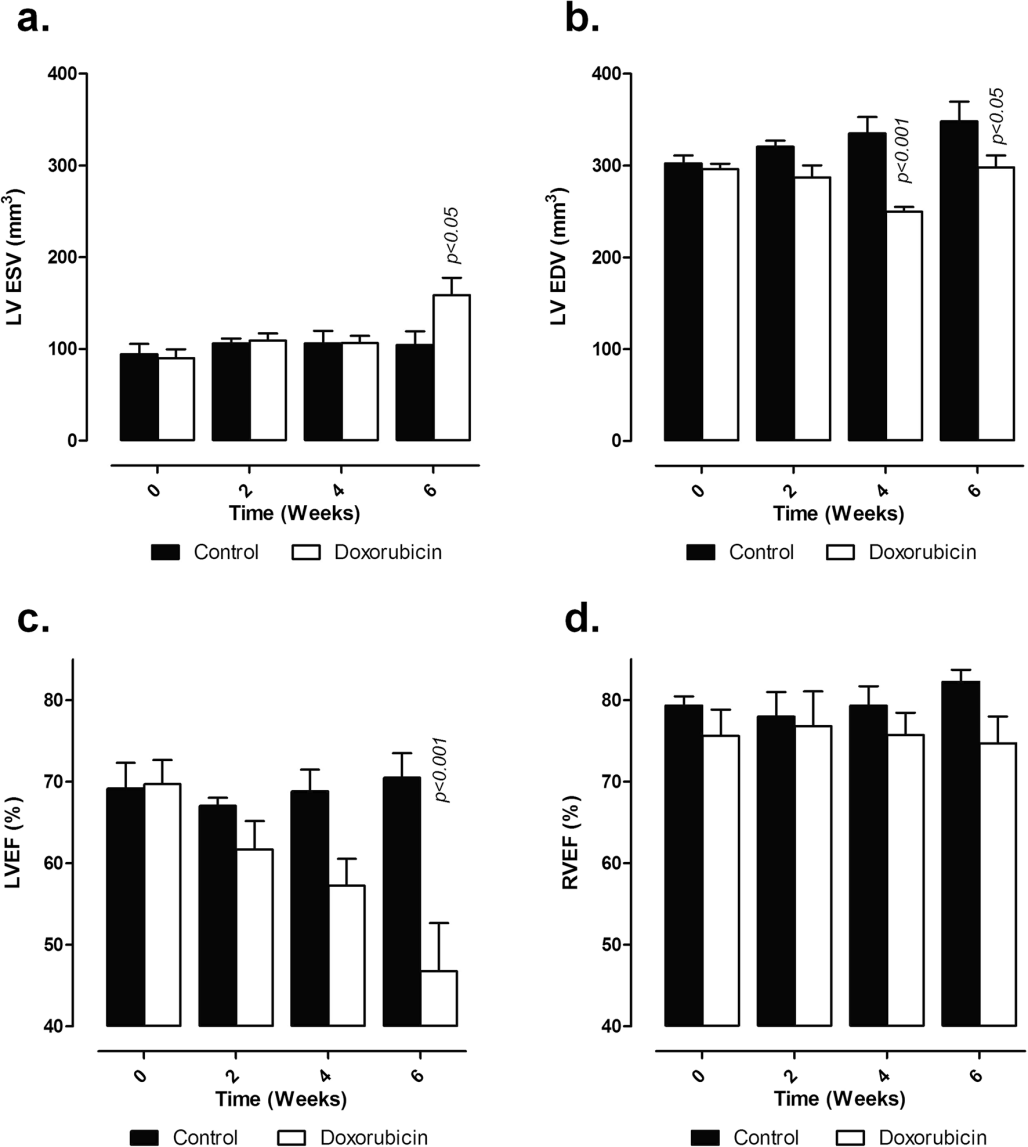
Cardiac function parameters measured every 2 weeks on cardiac cine MR images. **a** Left ventricle end-systolic volume; **b** left ventricle end-diastolic volume; **c**, **d**, respectively, left and right ventricle ejection fractions. *LV ESV* Left ventricle end-systolic volume, *LV EDV* left ventricle end-diastolic volume, *LVEF* left ventricle ejection fraction, *RVEF* right ventricle ejection fraction. Data are expressed as mean ± SEM

Cardiac function evaluated by MRI was clearly impacted by doxorubicin injections (Figs. 1 and 2). All parameters measured were similar in both experimental groups at baseline and week 2. In the doxorubicin group, LV ESV was unchanged at week 4, but significantly increased at week 6 (Fig. 2a). LV EDV was significantly reduced from week 4 onwards (250 ± 5 mm^3^ in doxorubicin group vs. 335 ± 18 mm^3^ in control group). This reduction was maintained at week 6 (298 ± 13 mm^3^ in doxorubicin group vs. 348 ± 21 mm^3^ in control group) (Fig. 2b). While there is no significant reduction of LV EF at week 4, the decrease in LV EDV combined with increased LV ESV resulted in a significant 33% reduction of LV EF at week 6 (47 ± 6% in doxorubicin group vs. 70 ± 3% in control group) (Fig. 2c). RVEF was not different between control and doxorubicin treated rats whatever the time point examined (Fig. 2d).

### Cardiac morphology

**Fig. 3:**
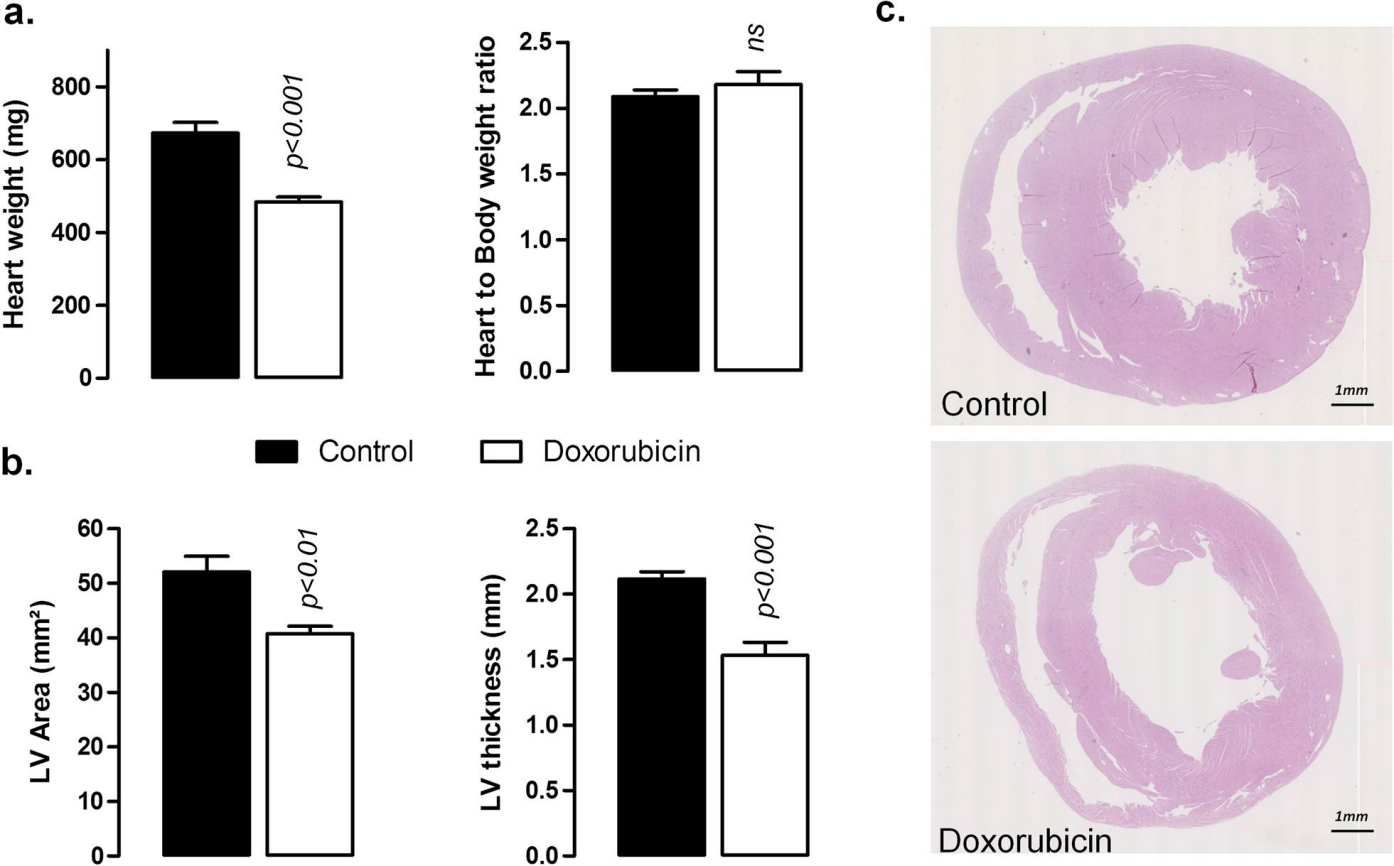
Evaluation of cardiac morphology at the end of the 6 weeks of experimental protocol. **a** Weight of the heart at the time of harvesting and heart-to-body weight ratio. **b** Histomorphometry measurements of left ventricular area and left ventricular thickness measured on Hematoxylin–eosin stained cardiac slices. *LV* Left ventricle. Data are expressed as mean ± SEM. **c** Representative full view of cardiac morphology of Hematoxylin–eosin stained cardiac slices in control (upper panel) and doxorubicin-treated rats (lower panel)

Cardiac morphology was evaluated using MR images to determine LV mass (Table 1), by weighing the heart at the time of euthanasia (Fig. 3a) and using histomorphometry on cardiac sections (Fig. 3b, c). LV mass was similar in vehicle and doxorubicin-treated rats at baseline. From week 2 and until week 6, LV mass was significantly reduced in the doxorubicin-treated group (Table 1). This result was confirmed by the weight of the heart (left and right ventricles) at week 6 with a mean weight of 673 ± 29 mg for the control group and 484 ± 13 mg in the doxorubicin group corresponding to a 28% lower weight (Fig. 3a). However, since body weight was also 30% lower in doxorubicin group, the heart to body weight ratio was similar between the vehicle and doxorubicin-treated rats (Fig. 3a). Histomorphometric measurements showed that concomitantly to the LV mass and heart weight reduction in the doxorubicin group compared with the control group, LV area was significantly decreased by 22% (52 ± 3mm^2^ in the control group and 41 ± 3mm^2^ in the doxorubicin group) (Fig. 3b, c). LV thickness was also reduced by the same extent (Fig. 3b).

### [^18^F]FDG cardiac uptake

**Fig. 4:**
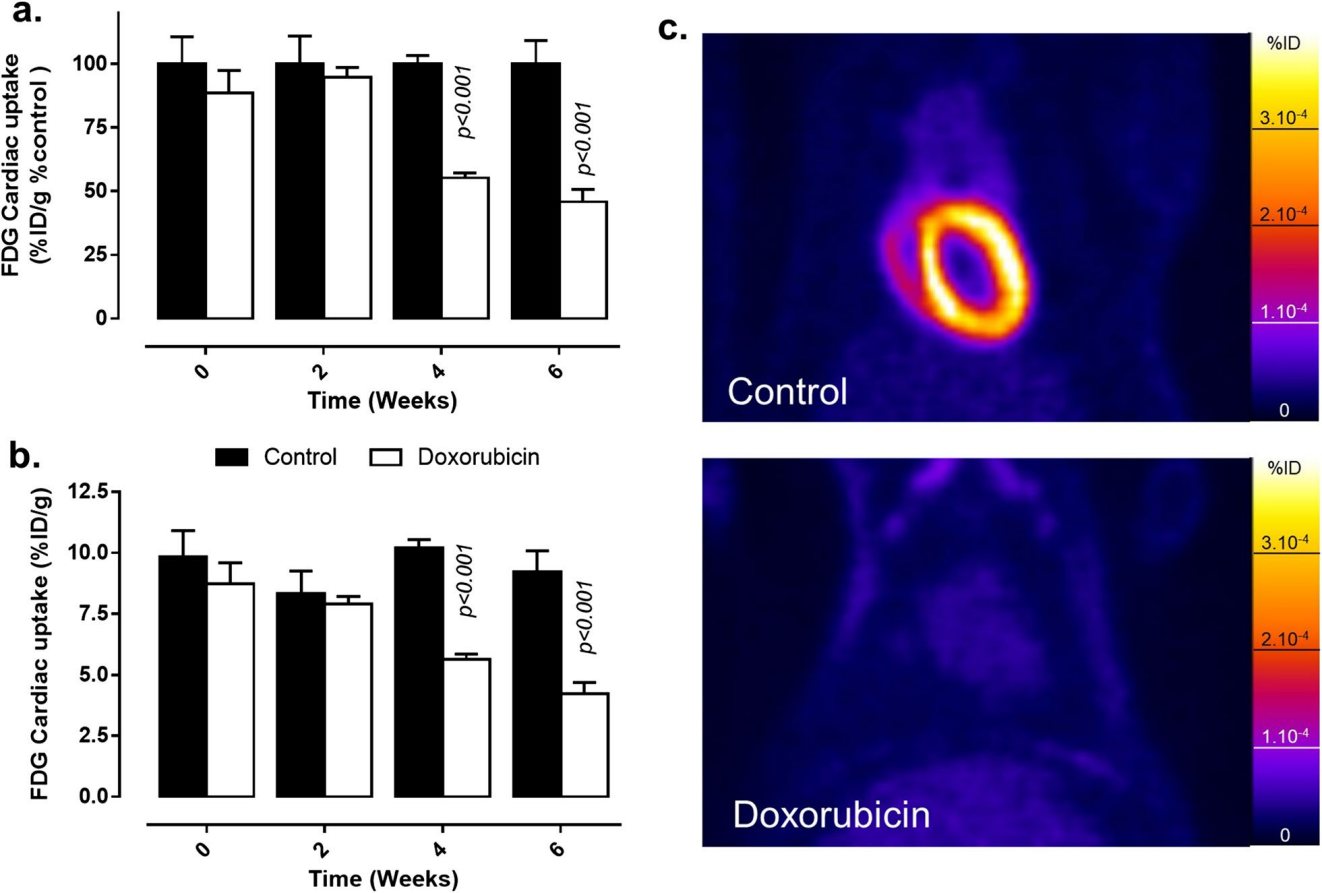
Evaluation of cardiac [^18^F]FDG uptake in vehicle and doxorubicin-treated rats measured on PET images. Cardiac [^18^F]FDG uptake monitoring during the 6 weeks of experimental protocol. Data are expressed as % ID/g related to control group (**a**) and %ID/g (**b**) and as mean ± SEM. *p* values presented on graphs are comparisons between control and doxorubicin groups **c**. Representative cardiac-centered coronal views of [^18^F]FDG PET images in control (upper panel) and doxorubicin-treated rats (lower panel) obtained at 6 weeks

Cardiac [^18^F]FDG uptake measured on PET images was similar in control and doxorubicin groups at baseline and week 2 (Fig. 4a, b; Additional file 1: Fig. S2). However, at weeks 4 and 6, cardiac uptake of [^18^F]FDG was significantly decreased by about 50% in the doxorubicin group compared with the control group (Fig. 4a, b) (respectively, 4.2 ± 0.5%ID/g and 9.2 ± 0.8%ID/g at week 6) while glycemia was unchanged (Table 1). The difference between experimental groups was clearly visible on PET images at week 6 (Fig. 4c). The results were similar when expressed as %ID (Additional file 1: Fig. S2).

Time-effect statistical analysis showed that while cardiac [^18^F]FDG was stable in control group from baseline to week 6 (*p* > 0.05 for all time points compared to baseline), It was significantly reduced in doxorubicin-treated rats at week 4 and 6 when compared to baseline (*p* < 0.001). Interestingly, [^18^F]FDG cardiac uptake correlated with LV ejection fraction with a correlation coefficient of 0.49 and *p* < 0.01 (Fig. 6a). It is to be noted that some data obtained at week 6 in doxorubicin treated-rats seemed to present low ^18^F]FDG cardiac uptake and normal LV ejection fraction (Fig. 6a). However, larger series would be necessary to determine if these specific cases are outliers or may constitute a subgroup of animals with a specific pattern of FDG uptake.

### [^123^I]MIBG cardiac uptake

**Fig. 5:**
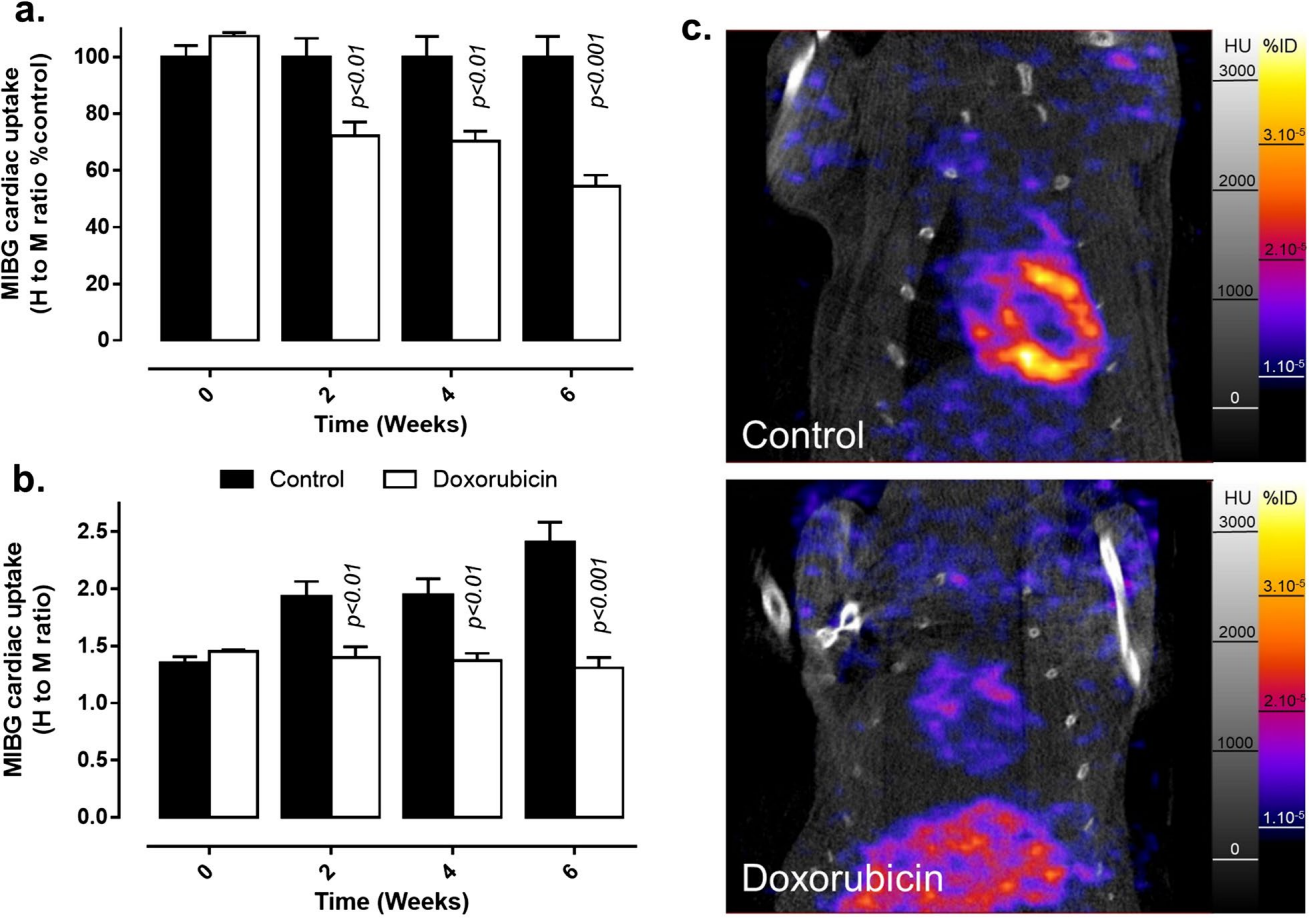
Evaluation of cardiac [^123^I]MIBG uptake in vehicle and doxorubicin-treated rats measured on SPECT images. **a**, **b** Cardiac [^123^I]MIBG uptake monitoring during the 6 weeks of experimental protocol. Data are expressed as heart to mediastinum (H to M) ratio related to control group (**a**) and H to M ratio (**b**) and as mean ± SEM. *p* values presented on graphs are comparisons between control and doxorubicin groups. **c** Representative cardiac-centered coronal views of [^123^I]MIBG SPECT-CT images in control (upper panel) and doxorubicin-treated rats (lower panel) obtained at 6 weeks

**Fig. 6:**
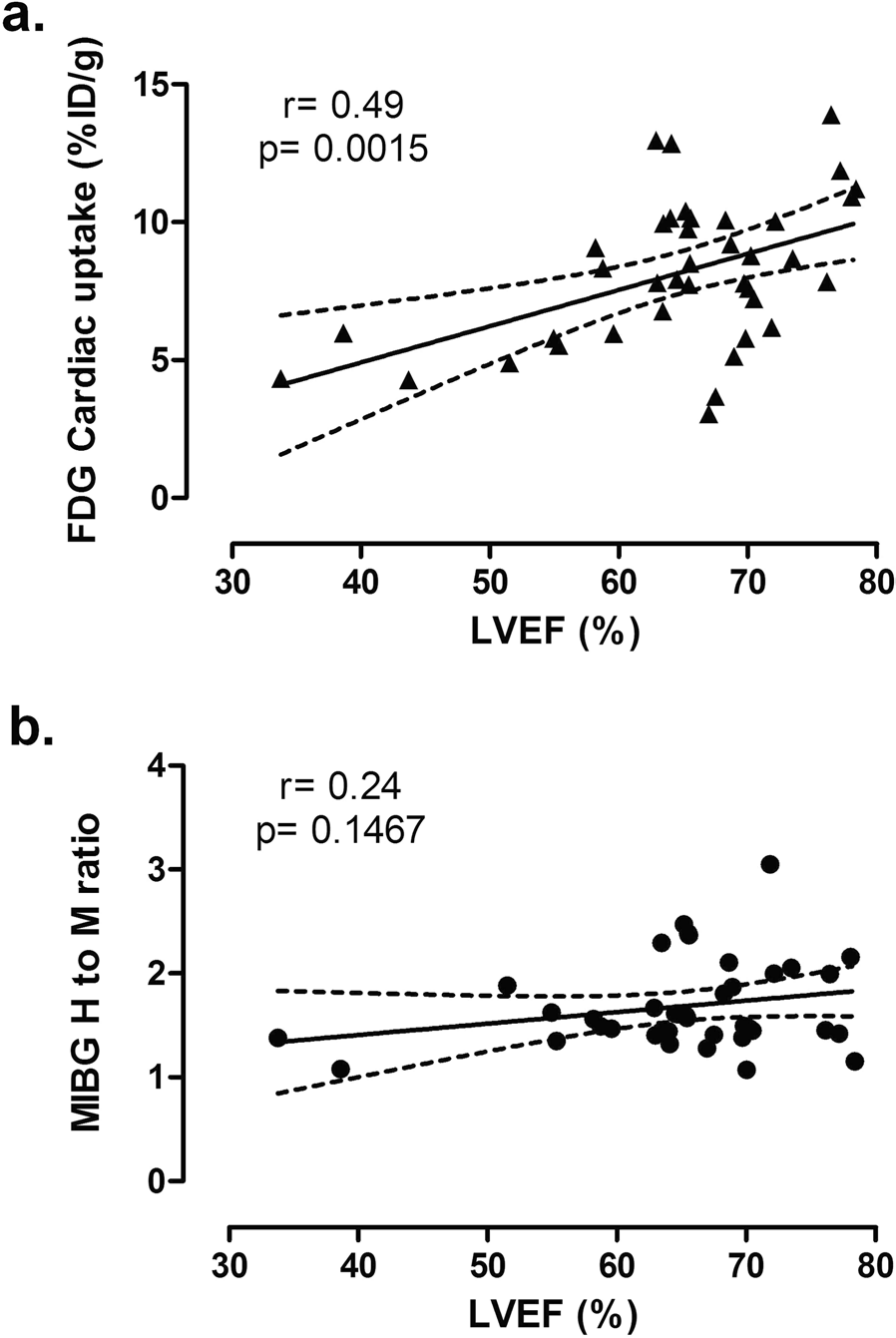
Correlation between LV ejection fraction and [^18^F]FDG cardiac uptake (**a**) or [^123^I]MIBG cardiac uptake (**b**). Data were obtained using Pearson correlation. Graphs represent linear regression line and 95% confidence interval (dotted line). H to M: heart to mediastinum

Cardiac [^123^I]MIBG H to M ratio monitoring showed a different pattern than cardiac function and [^18^F]FDG cardiac uptake. If baseline imaging showed equivalent [^123^I]MIBG H to M ratios between experimental groups, the [^123^I]MIBG H to M ratio was clearly lower in the doxorubicin group than in the control group from the 2 week timepoint onwards (Fig. 5a, b). This difference between experimental groups was maintained at 4 and 6 weeks (Fig. 5a–c). Similar statistical differences were observed between experimental groups when data in %ID are analyzed (Additional file 1: Fig. S3). As a consequence, the [^123^I]MIBG H to M ratio did not correlate with ejection fraction as shown on Fig. 6b (*p* > 0.05).

## Discussion

We showed that both glucose metabolism evaluated by [^18^F]FDG/PET and cardiac innervation evaluated by [^123^I]MIBG / SPECT were impacted by a doxorubicin repeated administration in rats. Our work constitutes the first longitudinal study evaluating simultaneously in the same animals, cardiac function, glucose metabolism and sympathetic innervation following anthracycline treatment. In addition, we showed that the modification on [^123^I]MIBG preceded the change in [^18^F]FDG uptake and cardiac dysfunction. Altogether, these results suggest that cardiac innervation imaging may constitute a better early marker of cardiac dysfunction associated with anthracycline therapy than glucose metabolism imaging.

Several studies previously examined that [^123^I]MIBG uptake changes after anthracycline administration in both human [8, 16–18] and animals [19–21]. In accordance with these data, we showed that the cardiac [^123^I]MIBG H to M ratio was markedly lower in doxorubicin treated rats. Moreover, cardiac [^123^I]MIBG monitoring showed a different pattern than cardiac function and [^18^F]FDG cardiac uptake with a clear decrease from the 2 week timepoint when compared to control group, before any modification of cardiac function parameters. Surprisingly, cardiac [^123^I]MIBG uptake regularly increased from baseline to week 6 in our control group. No clear explanation has been found in previously published studies. It can be however hypothesized that since the animals used in the present work were 6–8 weeks old at baseline, as for most of preclinical cardiovascular studies performed in rodents, they were still growing. This result emphasized the major role of carrying out a control group to be able to detect potential effects of a treatment. The mechanisms involved in the decrease of cardiac [^123^I]MIBG uptake are not fully understood. It was suggested that destruction of adrenergic nerve tissue caused by oxidative stress associated with doxorubicin administration could play a role [19]. However, further studies are needed to more thoroughly explore the mechanisms involved.

Moreover, we have shown that while [^18^F]FDG cardiac uptake correlated with LV ejection fraction, the [^123^I]MIBG H to M ratio did not. These results suggest that the modification of [^18^F]FDG cardiac uptake follows cardiac dysfunction impairment and therefore, do not constitute a valuable early marker of cardiotoxicity induced by anthracyclines. Indeed, the variability of baseline [^18^F]FDG cardiac uptake in clinical practice could also constitute an obstacle for a possible development of cardiac [^18^F]FDG PET imaging to evaluate cardiotoxicity of anticancer agents. Conversely, [^123^I]MIBG cardiac SPECT-CT could constitute a good alternative. Indeed, the absence of correlation between LV ejection fraction and [^123^I]MIBG uptake, tended to indicate that the time course of changes for these parameters were different, i.e. [^123^I]MIBG changes appeared before LV ejection fraction decrease. However, the routine use of [^123^I]MIBG cardiac SPECT-CT in clinical practice needs to be discussed. Indeed, quantification of the SPECT signal in clinical practice is delicate. However, the development of new Cadmium-Zinc-Telluride cameras could overcome this limitation thanks to the improved spatial resolution and quantification capabilities [22]. New PET tracers of cardiac innervation are also under evaluation [23]. Among the most advanced tracers under development, [^18^F]flubrobenguane may be the most promising [24, 25] with a minimal liver uptake [24], a good cardiac wall signal-to-noise ratio in both humans and animals [24, 25], and a stable storage in nerve terminals [25]. It would be therefore particularly interesting to evaluate this new tracer as an early marker of anticancer therapy cardiotoxicity, especially using PET/MR that could simultaneously evaluate cardiac function and morphology.

Similarly to numerous previously published preclinical studies, we reported impairment of cardiac function and morphology following anthracycline therapy [26–31]. However, it is quite delicate to compare studies because there is no consensus about how to induce cardiac dysfunction following doxorubicin administration in rodents. Indeed, administration routes (intravenous [9, 14, 19, 28], intraperitoneal [16, 29, 32, 33], or subcutaneous [26, 31]), treatment schedule and doses administered (from 5 to 50 mg/kg) varied widely between studies. In the present work, we choose to use intravenous injections of doxorubicin and to repeat the treatment 3 times at 10 days’ interval (15 mg/kg cumulative dose) to better mimic clinical practice. In our experimental conditions, doxorubicin treatment induced cardiac dysfunction with a significant reduction of ejection fraction after 6 weeks which was preceded by a decrease in end-diastolic volume from the 4 week-time point. If ejection fraction decrease is well described in the literature, data on diastolic function are scarce [34]. Indeed, most studies focused on ejection fraction and did not report changes of systolic or diastolic parameters. To our knowledge, the decrease in end-diastolic volume observed in our study has not been reported before. A close monitoring of diastolic function and volumes should be considered to better detect possible cardiotoxic events related to anthracyclines.

We also reported a significant decrease of cardiac [^18^F]FDG uptake in the doxorubicin group compared with the control group at weeks 4 and 6. While [^18^F]FDG PET has been suggested as a potential marker for anthracycline-induced cardiotoxicity, few studies have been performed, and results are conflicting. Most publications reported an increased cardiac [^18^F]FDG uptake after anthracycline administration [9, 10, 12, 14, 35, 36], but some results indicated a maintained or decreased [^18^F]FDG cardiac uptake [11, 13, 31]. The discrepancies between studies, and the results observed in our work, may have different explanations. The delay between last anthracycline administration and PET acquisition are not always precised [11, 12, 35, 36] and could rather reflect acute transient effects such as lipid peroxidation, inflammation or apoptosis [32, 33] than long-term effects. In most of the preclinical studies, the delay between last doxorubicin administration and [^18^F]FDG uptake administration is fixed between 6 days and 2 weeks [9, 13, 14, 31] excluding acute effect observation. The results reported were, however, not always in agreement. Surprisingly, [^18^F]FDG cardiac uptake levels in control animals varied considerably: when we report 9.8 ± 1.0% ID/g (Fig. 4), others observed 1.5 to 2% ID/g [16] or quite low values [9, 14]. In accordance with our study, a much higher cardiac uptake of [^18^F]FDG was reported by Shen et al*.* [13] with a baseline SUV of 9.4 ± 2.1 in control groups compared with 0.8 in the Bauckneht et al*.* study [14]. Mechanisms of action involved in the decrease in cardiac uptake of [^18^F]FDG were not evaluated here, but several hypotheses could be drawn. Firstly, experimental conditions could vary between studies [9, 13, 14, 31] with animals that can be fasted or not, different types of anesthetics were used, and uptake period could also be variable and performed in awake or anesthetized animals. There would be therefore a need for more precise guidelines for [^18^F]FDG cardiac PET imaging in preclinical studies. Secondly, [^18^F]FDG cardiac uptake could also be impacted by a decreased myocardial perfusion However, previously reported results [31] and internal data in another set of animals (Additional file 1: Table 1) showed that cardiac perfusion was not impacted by doxorubicin using [^99m^Tc]Sestamibi SPECT imaging. We actually showed that [^99m^Tc]Sestamibi cardiac uptake, which is a tracer of myocardial tissue perfusion, was similar in control and doxorubicin treated rats up to week 6 after the beginning of doxorubicin administration. A general reduction of myocardial metabolism constitutes thus a more probable explanation for our results as previously suggested by other authors [2, 31]. Finally, one limitation of the present work results from the possible partial volume effect on PET images associated with cardiac remodeling that could not be excluded to play a role since LV thickness was reduced from 2.1 to 1.5 mm corresponding therefore to the range in which this effect may exist.

## Conclusions

In conclusion, we showed that [^18^F]FDG cardiac uptake modifications after doxorubicin administration in rats were concomitant to cardiac dysfunction establishment. However, in the same set of animals, we demonstrated that the cardiac [^123^I]MIBG H to M ratio was modified earlier, before any signs of cardiac dysfunction. Thus, cardiac innervation imaging should be considered in the future to detect early cardiac toxicity of anticancer treatments. Further studies are nevertheless needed to explore new PET tracers of cardiac innervation and their potential to detect cardiac impact of other anticancer treatments such as VEGF blockers, tyrosine kinase inhibitors, immune checkpoint inhibitors and radiation therapy.

## Supplementary Information

**Additional file 1.** **Supplementary Fig S1.** Representative view of [^123^I]MIBG cardiac uptake image analysis method. **Supplementary Fig S2.** Evaluation of cardiac [^18^F]FDG uptake in vehicle and doxorubicin-treated rats measured on SPECT images expressed as %ID.** Supplementary Fig S3.** Evaluation of cardiac [^123^I]MIBG uptake in vehicle and doxorubicin-treated rats measured on SPECT images expressed as %ID. **Supplementary Table 1.** [^99m^Tc]- sestamibi cardiac uptake (%ID).

## Data Availability

The datasets analyzed during the current study are available from the corresponding author on reasonable request.

## Abbreviations

MR: Magnetic resonance
MIBG: Metaiodobenzylguanidine
FDG: Fluorodeoxyglucose
PET: Positron emission tomography
SPECT: Single photon emission computed tomography
CT: X-ray computed tomography
LV: Left ventricle
EDV: End-diastolic volume
ESV: End-systolic volume
EF: Ejection fraction
RVEF: Right ventricular ejection fraction
ID: Injected dose
H to M: Heart to mediastinum
