# Supplementary material for: [123I]MIBG is a better early marker of anthracycline cardiotoxicity than [18F]FDG: a preclinical SPECT/CT and simultaneous PET/MR study
Source: EJNMMI Res. 2021 Sep 20;11:92. doi: 10.1186/s13550-021-00835-1 (PMC8452816; doi:10.1186/s13550-021-00835-1)
Supplement: Supplementary file 1 — Additional file 1. Supplementary Fig S1. Representative view of [123I]MIBG cardiac uptake image analysis method. Supplementary Fig S2. Evaluation of cardiac [18F]FDG uptake in vehicle and doxorubicin-treated rats measured on SPECT images expressed as %ID. Supplementary Fig S3. Evaluation of cardiac [123I]MIBG uptake in vehicle and doxorubicin-treated rats measured on SPECT images expressed as %ID. Supplementary Table 1. [99mTc]- sestamibi cardiac uptake (%ID). [file 13550_2021_835_MOESM1_ESM.docx]

**Supplementary Information**

[^123^I]MIBG is a better early marker of anthracycline cardiotoxicity than [^18^F]FDG: a preclinical SPECT/CT and simultaneous PET/MR study

*A Oudot, A Courteau, M Guillemin, JM Vrigneaud, PM Walker, F Brunotte, A Cochet, B Collin*

**Supplementary Fig S1.** **Representative view of** **[^123^I]MIBG cardiac uptake image analysis method**

Representative SPECT-CT coronal image of cardiac [^123^I]MIBG uptake showing in white the delineation of cardiac region of interest and in green the mediastinal region of interest used for image analyses.

**
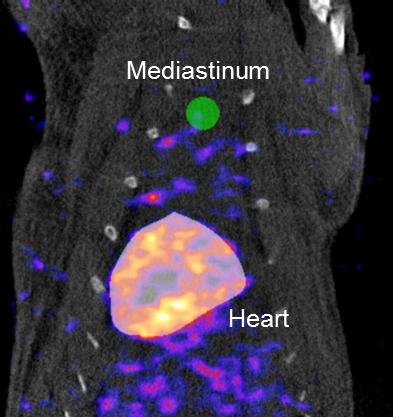
**

**Supplementary Fig S2. Evaluation of cardiac [^18^F]FDG uptake in vehicle and doxorubicin-treated rats measured on SPECT images.**

Cardiac [^18^F]FDG uptake monitoring during the 6 weeks of experimental protocol. Data are expressed as %ID and as mean ± SEM.

**Supplementary Fig S3. Evaluation of cardiac [^123^I]MIBG uptake in vehicle and doxorubicin-treated rats measured on SPECT images.**

Cardiac [^123^I]MIBG uptake monitoring during the 6 weeks of experimental protocol. Data are expressed as %ID and as mean ± SEM.

**Supplementary Table 1.** **[^99m^Tc]- sestamibi cardiac uptake (%ID)**

| % ID values | **Control** | **Doxorubicin** | ***P value*** |
| --- | --- | --- | --- |
| Baseline  Week 2  Week 4  Week 6 | 1.70 ± 0.11  1.45 ± 0.25  1.15 ± 0.19  1.11 ± 0.15 | 1.37 ± 0.16  1.18 ± 0.21  1.35 ± 0.15  1.16 ± 0.06 | *ns*  *ns*  *ns*  *ns* |

Quantifications were performed on SPECT-CT images after 150 MBq [^99m^Tc]-Sestamibi intravenous injection and corrected to injected dose corrected to radioactive decay. Experimental groups and treatments were similar than those described in the main manuscript. ns: not significant. Results are expressed as mean ± SEM.
